# Genomic epidemiology and antimicrobial resistance prevalence of *Staphylococcus aureus* isolated from bloodstream infections in a tertiary hospital in central China: a 5-year retrospective study

**DOI:** 10.3389/fpubh.2026.1807676

**Published:** 2026-04-14

**Authors:** Sudi Zhu, Kaixuan Zhang, Mengyu Zhang, Jianxiong Xiao, Lusheng Wang, Caixia Zhao

**Affiliations:** 1 Wanbei Coal-Electricity Group General Hospital, Suzhou, China; 2 Medical College of Qinghai University, Xining, China

**Keywords:** antibiotics, bloodstream infections, methicillin-resistant *Staphylococcus aureus*, molecular epidemiology, whole-genome sequencing

## Abstract

*Staphylococcus aureus* causes severe bloodstream infections (BSIs), and methicillin-resistant *S. aureus* (MRSA) poses a major clinical threat due to strong antimicrobial resistance and limited therapeutic options. This retrospective study analyzed 96 *S. aureus* isolates from nosocomial BSIs in a tertiary hospital in central China from 2020 to 2024, to clarify the epidemiological characteristics of antimicrobial resistance and transmission mechanisms. Antimicrobial susceptibility testing, whole-genome sequencing and bioinformatics analysis were performed to identify antibiotics c resistance genes (ARGs), virulence factors (VFs), multilocus sequence typing (MLST), staphylococcal cassette chromosome *mec* (SCCmec) typing and phylogenetic features. MRSA accounted for 72.92% (70/96) of all strains. The total number of *S. aureus* isolates trended downward, while the MRSA proportion remained stable at 57.14–100.00%, with the Department of Nephrology showing the highest infection rate (36.46%). All strains were susceptible to linezolid and rifampicin; one vancomycin-resistant, one tigecycline-resistant and one phenotypically discordant MRSA strain were detected. Genomic analysis identified 29 resistance gene types, with frequent co-carriage of *mec* and *bla* family genes. Strains carried abundant VFs (average 74 genes per strain), including 45 core virulence genes and widely distributed *lukF-PV*/*lukS-PV*. MLST revealed 21 sequence types, dominated by ST59 (21.88%) and persistently epidemic ST22. SCCmec type IV (58.93%) was predominant, with no *mecI* in type IV/V strains and three pseudo-SCCmec carriers. Genomic epidemiology provides reliable evidence for precise medication, targeted prevention and control of MRSA BSIs, and references for understanding *S. aureus* antimicrobial resistance evolution.

## 1 Introduction

Bloodstream infection (BSI) is a severe infectious syndrome characterized by rapid progression and high mortality, which can be caused by a variety of pathogens. Among them, methicillin-resistant *Staphylococcus aureus* (MRSA) is one of the major microorganisms leading to this syndrome, and the incidence of community-acquired MRSA (CA-MRSA) infections continues to rise (1). As an important subtype of *S. aureus*, MRSA is defined by its resistance to β-lactam antibiotics, which limits therapeutic options and is associated with prolonged hospital stays and increased medical costs (2). MRSA possesses strong abilities of adhesion, invasion, and immune evasion; it can rapidly progress from local infections or bacteremia without obvious foci to sepsis, septic shock, and even multiple organ dysfunction syndrome (MODS) (3). Adverse outcomes are particularly common in critically ill patients, those undergoing invasive procedures, immunocompromised individuals, and patients with multiple comorbidities (4). Clinically, MRSA in BSIs usually requires treatment with glycopeptides, oxazolidinones, or other alternative agents. Additionally, special considerations such as patients’ renal insufficiency, drug-related adverse reactions, and potential drug–drug interactions need to be taken into account, resulting in a narrower therapeutic window (5, 6). Furthermore, the multidrug-resistant phenotype, i.e., the coexistence of multiple resistance-related genes, may further increase the risks of treatment failure and recurrence (7). Beyond its impact on individual prognosis, MRSA can also persistently transmit or cause clustered outbreaks in healthcare settings (8), making it not only a long-standing challenge in clinical infectious diseases but also a continuous threat to infection prevention and antimicrobial stewardship.

At the population level, the dominant clone lineages, antimicrobial resistance profiles, and pathogenicity of MRSA can exhibit patterns of clone replacement and coexistence across different countries, regions, and even among different hospitals within the same region (9). A genomic analysis of 405 global ST88 *S. aureus* isolates (including 52 MRSA strains) in 2024 showed that ST88-MRSA isolates from BSIs originating in China (especially the Clade I branch) have embarked on an independent evolutionary path since approximately 1964, followed by significant clonal dissemination. These strains carry unique SCCmec IVc and pseudo-SCCmec elements, while retaining the intact *sraP* adhesion gene associated with enhanced biofilm-forming ability, confirming the risk of interregional transmission of MRSA strains (10). Studies on clinical isolates from Macao between 2017 and 2022 demonstrated a significant increase in the detection rate of MRSA in BSIs, rising from 30.1 to 45.7%, with SCCmec type IV as the predominant type; however, these strains remained fully susceptible to key drugs such as vancomycin and linezolid (11). The transmission of MRSA is associated with adaptive evolution, including the diversification of antimicrobial resistance determinants, the acquisition or loss of mobile genetic elements, and changes in pathogenicity driven by differences in virulence factor combinations (12, 13). For nosocomial transmission of MRSA, it often occurs in an occult manner, shaped by factors such as ward structure, patient flow, use of invasive devices, antimicrobial selective pressure, and insufficient infection prevention (14, 15). Therefore, identifying local dominant clones, recognizing potential clustering and transmission links, and clarifying the genetic basis of antimicrobial resistance are crucial for guiding empiric therapy, formulating intervention strategies, and evaluating prevention and control efficacy.

Traditional molecular typing methods such as pulsed-field gel electrophoresis (PFGE), multilocus sequence typing (MLST), and *spa* typing have played important roles in MRSA surveillance and preliminary source tracing (16). However, these traditional molecular typing methods fail to accurately distinguish isolates with extremely close genetic distances over short time scales and cannot provide comprehensive information on antimicrobial resistance and virulence determinants. Genomic epidemiology based on whole-genome sequencing (WGS) can detect subtle variations across the entire genome; when combined with core genome phylogenetic analysis, WGS enables more precise resolution of population structure and clonal clustering, as well as inference of potential transmission links. Furthermore, WGS can provide information on antimicrobial resistance determinants and related mutations, offering molecular evidence to explain resistant phenotypes and guide antimicrobial selection (17, 18). Integration of WGS with epidemiological data can promptly identify nosocomial transmission chains and evaluate the effectiveness of infection control measures (19).

The present study focuses on *S. aureus* isolates from BSIs consecutively collected over a five-year period (2020–2024) in a tertiary hospital in central China. Adopting a retrospective design, this study integrates WGS data, clinical information, and epidemiological data to clarify the dominant MRSA clone lineages, annual variations, and dynamic changes in MRSA proportions among BSI isolates in the hospital, identify transmission clusters and high-risk departments, and elucidate the antimicrobial resistance gene profiles of MRSA from BSIs and their associations. This study aims to provide core evidence for similar medical institutions to establish genomic surveillance frameworks and address relevant clinical and public health challenges.

## 2 Materials and methods

### 2.1 Strain source, isolation, and identification

This was a single-center retrospective study. All blood culture-positive *S. aureus* isolates from BSI were consecutively included from January 2020 to December 2024. To minimize potential bias in epidemiological analyses, only the first isolate from each patient per hospitalization episode was included. These isolates were isolated, cultured, and purified according to the routine procedures of the clinical microbiology laboratory at a tertiary sentinel hospital in central China. Nosocomial BSI was defined according to the criteria established by the U.S. Centers for Disease Control and Prevention (CDC), which requires that the blood culture specimen be collected more than 48 h after hospital admission, with no evidence indicating that the infection was present or incubating at the time of admission. Species identification was performed using matrix-assisted laser desorption/ionization time-of-flight mass spectrometry (MALDI-TOF MS; Bruker BioSciences Corporation, Germany) and confirmed with the Biotyper 2.0 database. After re-verification and purification, strains were preserved in glycerol stocks at −80 °C for future use.

### 2.2 Antimicrobial susceptibility testing (AST) and MRSA determination

AST was conducted using the VITEK-2XL Compact automated microbial identification and susceptibility system (BioMérieux, France), with confirmation via AST-GP639 cards (BioMérieux, France). The antimicrobial test panel included linezolid, penicillin G, oxacillin, cefoxitin (screening test), levofloxacin, moxifloxacin, vancomycin, gentamicin, erythromycin, clindamycin (including the inducible clindamycin resistance test, i.e., “*D*-test”), trimethoprim-sulfamethoxazole, tigecycline, and rifampicin. AST results were interpreted in accordance with the 2025 edition of the Clinical and Laboratory Standards Institute (CLSI) M100 document. For tigecycline, for which CLSI does not provide interpretive breakpoints, susceptibility was interpreted according to the European Committee on Antimicrobial Susceptibility Testing (EUCAST) version 13.0 breakpoints (*S. aureus*, MIC ≤ 0.5 mg/L defined as susceptible), a standard that aligns with the intrinsic interpretation logic of the VITEK-2 XL system. For any isolates flagged as resistant to vancomycin or tigecycline by the automated system, the results were confirmed using the broth microdilution (BMD) method, with procedures and interpretation strictly following CLSI M100 guidelines. Phenotypic determination of MRSA was defined as an oxacillin minimum inhibitory concentration (MIC) ≥ 4 μg/mL or a positive cefoxitin screening test. For rare or discordant phenotypic results (e.g., oxacillin-susceptible but cefoxitin-screen-positive), re-verification was performed using the oxacillin agar screen test [Mueller-Hinton agar (MHA) plates containing 4% NaCl and 6 μg/mL oxacillin].

### 2.3 Genomic DNA extraction, library construction, and next-generation sequencing

After resuscitation, strains were streaked onto tryptic soy agar (TSA; Shandong Top Bio Engineering Co., Ltd., China) plates and cultured at 37 °C for 18–24 h. Single colonies were picked for purification and culture. Bacterial cells in the logarithmic growth phase were collected, and genomic DNA was extracted using the Coolaber Bacterial Genomic DNA Extraction Kit (cat. no. DE232; Shandong Top Bio Engineering Co., Ltd., China). DNA concentration was quantified with a Qubit 4.0 Fluorometer (Thermo Fisher Scientific, USA), and purity was assessed using a NanoDrop One/OneC spectrophotometer (Thermo Fisher Scientific, USA). Library construction was performed using the Illumina Nextera DNA Flex Library Prep Kit (Illumina, USA), followed by DNA fragmentation, end repair, A-tailing, adapter ligation, and PCR amplification. Sequencing was carried out on the Illumina NovaSeq 6000 platform using paired-end sequencing (PE150) mode.

### 2.4 Raw data quality control, assembly, and genomic annotation

After converting raw sequencing data to FASTQ format via base calling, quality assessment was performed using FastQC v0.11.9.^1^ Quality control and filtering were then conducted with fastp v0.23.2^2^ using the following parameters: remove adapter sequences, discard reads with N content >5%, trim bases with quality score <20, and truncate reads shorter than 50 bp. High-quality reads that passed quality control were used for *de novo* genome assembly with SPAdes v3.15.5 software. The careful parameter was set to reduce mismatches, and k-mer lengths suitable for bacterial genomes (e.g., 21, 33, 55, 77) were selected (20). Assembly quality was evaluated using QUAST v5.0.2,^3^ with metrics including total length, N50, number of contigs, and GC content. Assembly quality inclusion criteria were: N50 ≥ 20 kb, number of contigs ≤500, and total length consistent with the expected range of the *S. aureus* genome. CheckM^4^ was used to assess genome completeness and contamination, with quality control requirements of completeness ≥95% and contamination ≤5%.

Genomic annotation was performed using Prokka v1.14.6 (21), with default parameters combined with the NCBI RefSeq database for coding gene prediction, non-coding RNA identification, and functional annotation. For additional annotation of antimicrobial resistance genes and virulence factors, ABRicate v1.0.1^5^ was used to align against the Resfinder (22) and VFDB (23) databases.

### 2.5 MLST typing and phylogenetic tree construction

Based on WGS data, MLST was performed for all strains using mlst v2.23.0.^6^ *Staphylococcus aureus* ASM1342v1 was selected as the reference genome, and Snippy-core^7^ combined with Gubbins (24) was used to generate a recombination-corrected core genome. Phylogenetic tree construction was conducted using FastTreeMP^8^ with the GTRGAMMA model. The resulting tree file was visualized and optimized using Chiplot (25).

### 2.6 SCCmec typing of MRSA strains

Further SCCmec typing was performed for MRSA strains using SCCmecFinder v1.2 (26) with the 2024_01 database version to identify the mec complex and ccr gene type.

### 2.7 Ethics and data availability

De-identified clinical and strain data were used in this study. The study protocol was approved by the Ethics Committee of Wanbei Coal Electric Group General Hospital [Approval number: (WBCEGGH20241025033)], and a waiver of informed consent was granted. Raw whole-genome sequencing data have been submitted to the NCBI database under the BioProject Accession number PRJNA1419524.

## 3 Results

### 3.1 Nosocomial prevalence of *S. aureus* isolates from BSIs

**Figure 1:**
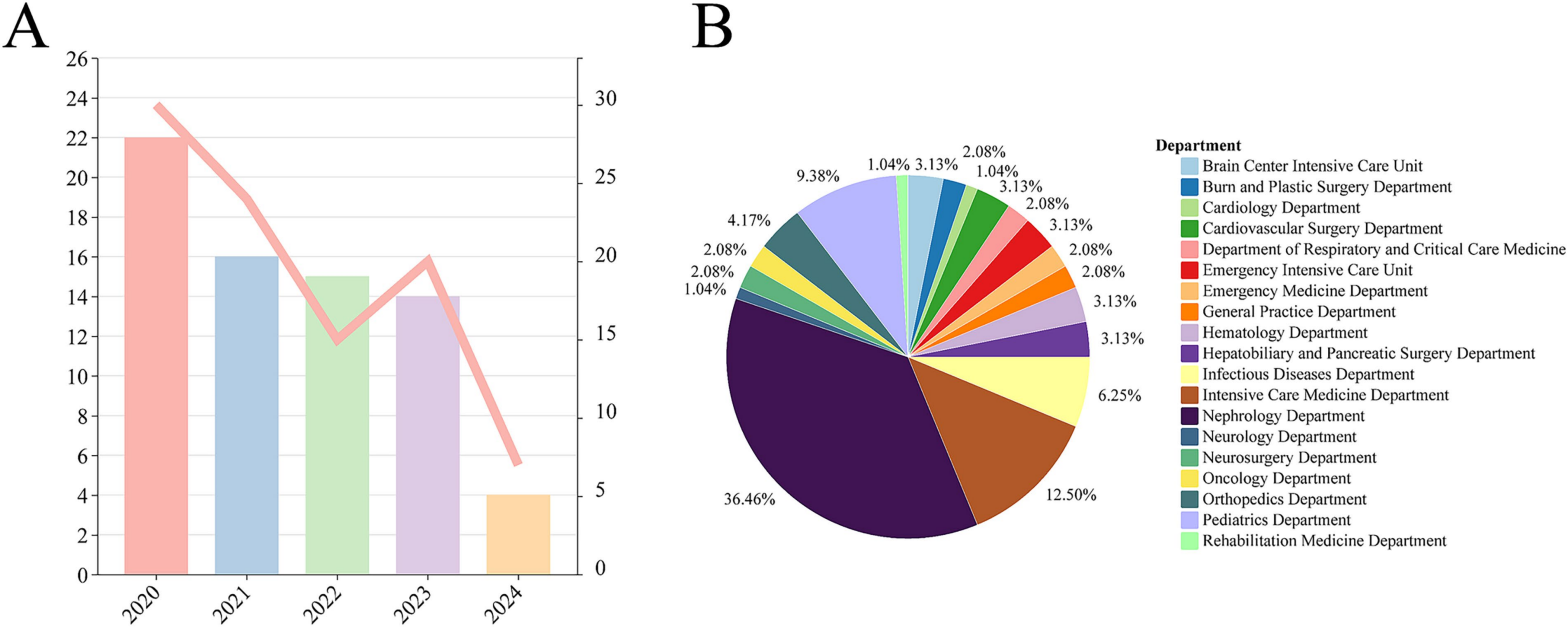
Nosocomial prevalence of *S. aureus*. **(A)** Columns represent the changes in the number of *S. aureus* isolates from 2020 to 2024, with reference to the left ordinate; the green line represents the annual number of *MRSA* isolates, with reference to the right ordinate. **(B)** Proportion of *S. aureus* isolates distributed across different departments from 2020 to 2024.

As described in the Methods, a total of 96 *S. aureus* strains were collected from a tertiary hospital in central China between 2020 and 2024, including 71 MRSA strains. As shown in Figure 1A, the detection rate of *S. aureus* from BSIs was the highest in 2020 (*n* = 30), followed by a gradual annual decline with a slight rebound in 2023 (*n* = 20), and only 7 strains were detected in 2024. Similarly, the number of MRSA isolates also showed a yearly decreasing trend, with the highest detection in 2020 (*n* = 22), accounting for 73.33% (22/30) of *S. aureus* isolates in the same year. Notably, the proportion of MRSA remained high during 2022–2023: 66.67% (14/24) in 2021, 100.00% (15/15) in 2022, 70.00% (14/20) in 2023, and 57.14% (4/7) in 2024. *S. aureus* was detected in 19 departments across the hospital, with a wide distribution. The Department of Nephrology had the most severe prevalence, accounting for 36.46% (*n* = 35) of all isolates, followed by the Department of Intensive Care Medicine (12.50%, 12/96) and the Department of Pediatrics (9.38%, 9/96).

### 3.2 Antimicrobial susceptibility phenotypes of *S. aureus* isolates from BSIs

After automated antimicrobial susceptibility testing and disk diffusion confirmation, the overall resistance level of *S. aureus* from BSIs was relatively low. Detailed susceptibility results are provided in Supplementary Table S1. As described in the Methods, oxacillin and cefoxitin screening tests identified 70 *S. aureus* strains resistant to oxacillin and 71 strains positive in the cefoxitin screen. Strain A215-20 showed conflicting results (oxacillin-susceptible but cefoxitin-resistant) and was confirmed as MRSA by the oxacillin agar screen test. Thus, a total of 71 MRSA strains were obtained, accounting for 72.92% of all *S. aureus* isolates from BSIs. All *S. aureus* strains in this study were susceptible to linezolid and rifampin. Among the 25 MSSA isolates, resistance to penicillin G was 92.00% (23/25), while susceptibility to both levofloxacin and moxifloxacin was observed in all isolates. Resistance to gentamicin was 12.00% (3/25). For macrolide–lincosamide antibiotics, resistance rates to erythromycin and clindamycin were both 60.00% (15/25). Additionally, resistance to trimethoprim/sulfamethoxazole was detected in 16.00% (4/25) of MSSA isolates. Among the 71 MRSA isolates, resistance to penicillin G was 100.00% (71/71). Resistance to levofloxacin was 19.72% (14/71), and to moxifloxacin was 11.27% (8/71), with 4.23% (3/71) showing intermediate resistance to moxifloxacin. Resistance to gentamicin was observed in 8.45% (6/71) of MRSA strains, including one strain with intermediate resistance. For macrolide–lincosamide antibiotics, resistance rates to erythromycin and clindamycin were 71.83% (51/71) and 69.01% (49/71), respectively, with one strain exhibiting intermediate resistance to clindamycin. Resistance to trimethoprim/sulfamethoxazole was 8.45% (6/71) among MRSA isolates. Notably, among these 71 MRSA strains, A12-22 isolated from the Department of Hepatobiliary and Pancreatic Surgery was resistant to vancomycin, and A200-20 isolated from the Department of Oncology was resistant to tigecycline. This phenomenon indicates that the prevention and control of MRSA face significant challenges.

### 3.3 Genomic characteristics of *S. aureus* isolates from BSIs

**Figure 2:**
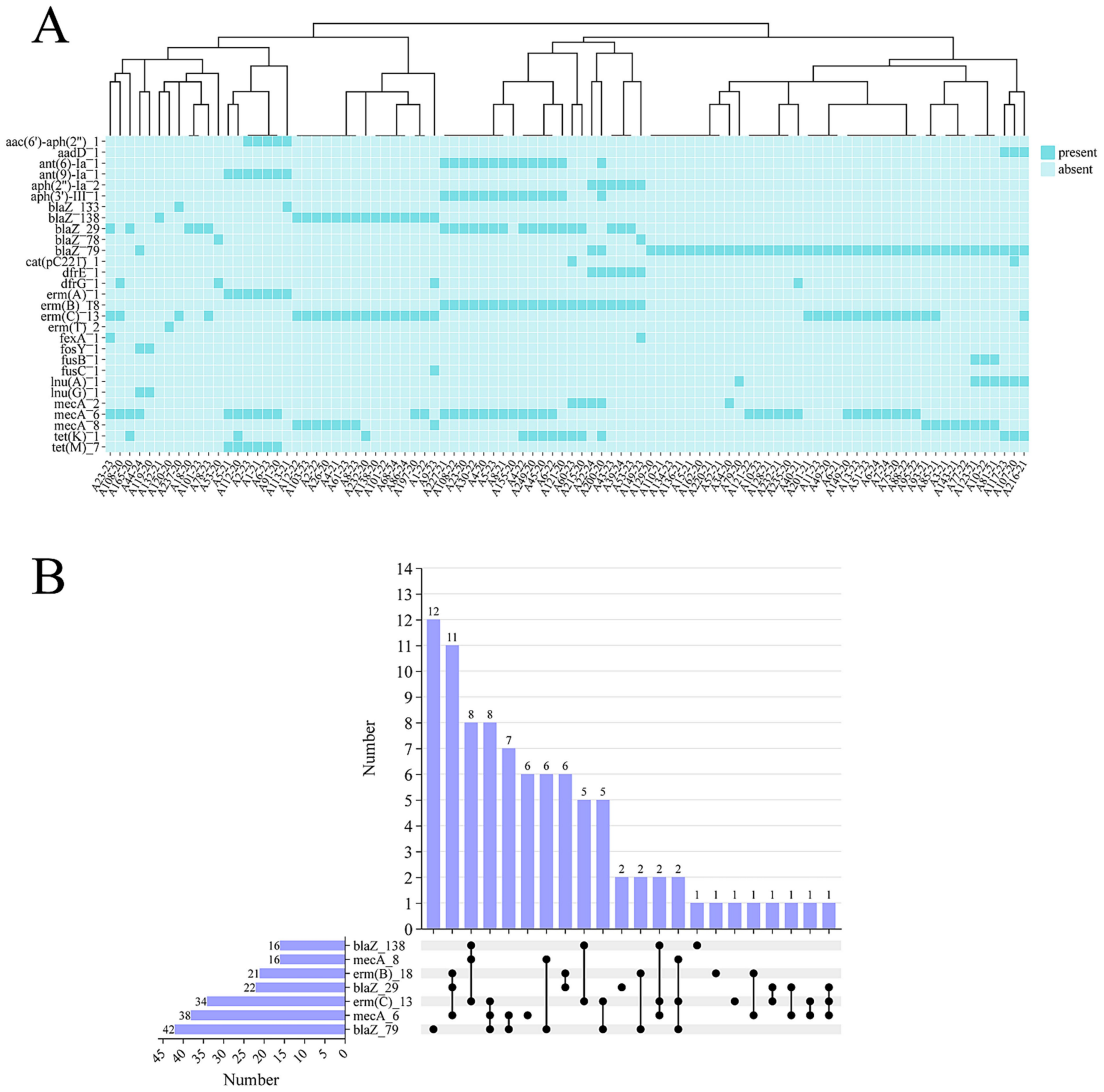
Epidemiological characteristics of antimicrobial resistance genes in *S. aureus* isolates from BSIs. **(A)** Clustering heatmap of presence/absence of antimicrobial resistance genes. **(B)** Upset plot showing the co-occurrence analysis of the top 7 most prevalent antimicrobial resistance genes.

Annotation using Resfinder identified 29 types of antimicrobial resistance genes in the 96 *S. aureus* isolates from BSIs, with an average of 3 resistance genes per strain (Supplementary Table S2). The most frequently detected resistance gene was *blaZ*_79 (*n* = 42), followed by *mecA*_6 (*n* = 38) and *erm* (C)_13 (*n* = 34), which confer resistance to penicillins, methicillin, and macrolide–lincosamide antibiotics, respectively (Figure 2A). Co-occurrence analysis of the top 7 most prevalent resistance genes (Figure 2B) showed that *blaZ*_79 was often present alone in *S. aureus* (*n* = 12). The second most common resistance gene combination was *mecA*_6/*blaZ*_29/*erm* (C)_18 (*n* = 11), followed by *erm* (C)_13/*mecA*_8/*blaZ*_138 and *erm* (C)_13/*mecA*_6/*blaZ*_79, each detected in 8 strains.

Meanwhile, virulence gene detection based on the VFDB database revealed a high abundance of virulence genes, with an average of 74 virulence genes per strain (Supplementary Table S3). Forty-five virulence genes associated with adhesion and biofilm formation (e.g., *fnb*, *ica*, *sdr*, *clf*, *ebp*, *atl*), immune evasion and host interaction (e.g., *cap*, *spa*, *eap*/*map*, *isd*, *ads*, *aur*, *esa*, *esx*), toxins (e.g., *hlg*, *hlb*, *luk*, *seb*, *eta*, *ssp*), and enzymes (e.g., *geh*, *hys*, *lip*, *srt*) were present in all 96 *S. aureus* strains. In addition, the leukocidins *lukS*-PV (*n* = 13) and *lukF*-PV (*n* = 57), which are closely associated with CA-MRSA infections, were widely detected. Furthermore, virulence genes related to severe or special infections, such as *tsst*-1 (*n* = 11), *eta*/*etb* (*n* = 1), and *sea*/*seb* (*n* = 25), were also identified. Collectively, these results indicate that *S. aureus* from BSIs not only possesses strong pathogenic potential but also carries multiple resistance genes that limit clinical treatment options, highlighting the necessity of monitoring the prevalence and transmission of this pathogen in hospitals.

### 3.4 Nosocomial lineage characteristics and population structure of *S. aureus* isolates from BSIs

**Figure 3:**
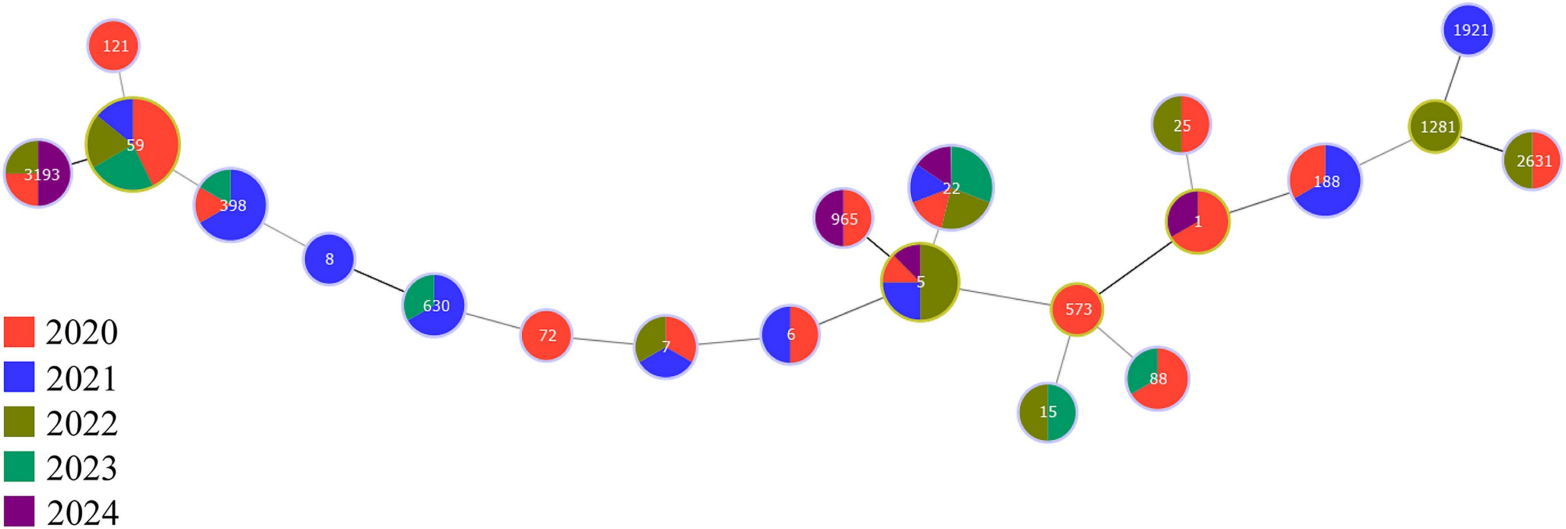
Minimum spanning tree (MST) based on MLST of *S. aureus* isolates from BSIs in different years.

MLST typing of the 96 *S. aureus* isolates from BSIs identified 21 sequence types (STs), including 10 unknown STs (Supplementary Table S4). ST59 (*n* = 21) was the dominant ST, followed by ST22 (*n* = 13) and ST5 (*n* = 8). A minimum spanning tree was constructed to illustrate the annual prevalence of STs (Figure 3). ST22 persisted and prevailed in the hospital over the 5-year period, with detection in each year. As the dominant lineage, ST59 was most prevalent in 2020 but not detected in 2024. Neither ST5 nor ST3191 was detected in 2023, while other *S. aureus* lineages occurred sporadically in different years. These results indicate that different lineages of *S. aureus* from BSIs exhibit temporal prevalence specificity. Furthermore, a brief analysis of the association between major STs and common resistance phenotypes was conducted. The prevalence of MRSA among ST59 and ST22 clones was 100% (21/21) and 100% (13/13), respectively. Resistance to erythromycin and clindamycin was highly prevalent in both ST59 (90.5 and 85.7%, respectively) and ST22 (100% for both). Levofloxacin resistance was more common in ST59 (33.3%) than in ST22 (7.7%). While all ST5 clones were MRSA, they exhibited relatively lower resistance rates to erythromycin, clindamycin, and levofloxacin (37.5, 37.5, and 0%, respectively).

**Figure 4:**
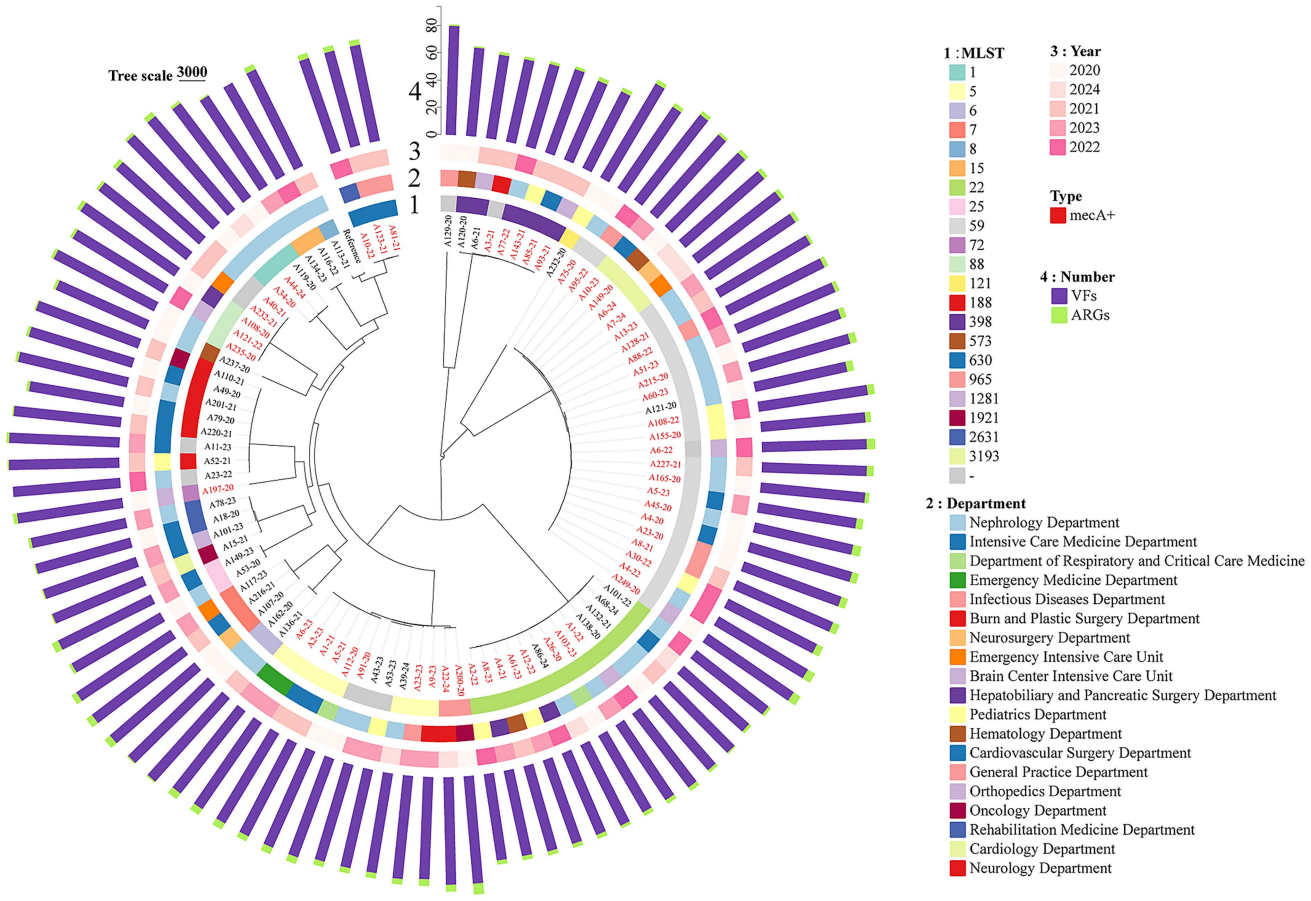
Phylogenetic tree of bloodstream-derived *S. aureus* constructed based on core genome variation.

To gain a more precise understanding of the population structure, a phylogenetic tree was constructed based on the recombination-corrected core genome (Figure 4). The population structure revealed by the phylogenetic tree was consistent with MLST typing, showing a good clustering pattern. ST5 and ST59 strains were mostly MRSA, and MRSA showed no significant correlation with department of origin or isolation year, presenting a sporadic distribution. MRSA strains often clustered together, indicating close genetic relatedness. The resistance and virulence genes carried by all *S. aureus* strains also showed no significant association with their origin or isolation time, reflecting the genotypic stability of *S. aureus* from BSIs.

### 3.5 SCCmec types and structures of *mecA*-positive *S. aureus* isolates from BSIs

As mentioned above, a total of 59 *mecA*-positive *S. aureus* strains were identified by Resfinder, among which *mecA*_6 (*n* = 38) was the most common, followed by *mecA*_2 (*n* = 5) and *mecA*_8 (*n* = 16). SCCmec typing for *mecA* structural subtyping (Supplementary Table S5) showed that type IV (58.93%, 33/59) was the predominant SCCmec type, followed by type V (22.03%, 13/59) and type II (11.86%, 7/59). Additionally, 3 strains carried pseudo-SCCmec elements, which may be novel or defective SCCmec elements. Each type exhibited distinct structural characteristics: all 7 type II strains belonged to subtype IIa and carried the complete mec gene complex (*mecI*-*mecR1*-*mecA*), while all type IV and V strains lacked the *mecI* gene and retained only the *mecR1*-*mecA* structure. Notably, 11 of the type IV strains (33.3% of type IV strains) were designated as “multiple” subtypes, suggesting that their structures may be variants or novel recombinant elements between known subtypes such as IVa and IVn. These results indicate that *mecA*-positive *S. aureus* in this study is dominated by community-acquired types (types IV and V), with a certain proportion of structural variants, providing key molecular evidence for further tracking clonal transmission and evolution.

## 4 Discussion

Based on single-center retrospective data from 2020 to 2024, this study systematically analyzed the epidemiological characteristics, antimicrobial resistance genomic patterns, clonal lineage evolution, and virulence gene profiles of *S. aureus* isolates from BSIs in a tertiary hospital in central China by integrating WGS data with clinical and epidemiological information. This study identified the core population structure with ST59 as the dominant clone and SCCmec type IV as the major resistance element, revealed the co-carriage characteristics of resistance genes and virulence genes, and simultaneously detected special clinical risk strains such as vancomycin-resistant strains, tigecycline-resistant strains, and discordant phenotypic strains. Notably, despite a persistently high MRSA proportion, the total number of *S. aureus* isolates from BSIs showed an overall declining trend from 2020 to 2024, reaching the lowest level in 2024. This trend may be associated with strengthened infection control measures and the implementation of antimicrobial stewardship programs, which have reduced inappropriate use of broad-spectrum antibiotics. The absence of the previously dominant ST59 clone in 2024 may indicate a temporary interruption in local clonal transmission or an ongoing shift in the predominant lineage. However, given the single-center retrospective design and limited sample size of this study, the specific drivers behind this decline and its generalizability require further validation through larger-scale, multicenter prospective studies. These findings provide direct molecular epidemiological and clinical evidence for precise prevention and control of regional MRSA infections, optimization of clinical medication, and construction of genomic surveillance systems, while supplementing the baseline epidemiological data on the infiltration of CA-MRSA into healthcare settings in central China.

This study found that although the overall detection number of *S. aureus* from BSIs showed a downward trend, the proportion of MRSA remained persistently high (57.14–100.00%, average 72.92%). This result indicates that while existing infection control measures can effectively reduce the overall infection rate of *S. aureus*, targeted prevention and control measures for MRSA remain inadequate. This phenomenon also suggests that MRSA can maintain its pathogenic advantage under sustained antimicrobial selective pressure due to its stronger environmental adaptability, antimicrobial resistance, and biofilm-forming ability. This finding is consistent with the results of Tang et al. in Xiangyang, who also pointed out that MRSA is more likely to be detected in BSIs (accounting for 55.6% of blood specimens) due to its significant resistance and biofilm properties (27). From a temporal perspective, the proportion of MRSA reached 100% (15/15) in 2022. Although limited by the sample size, combined with MLST typing results, it is speculated that there may have been local clustered transmission of the ST59-MRSA clone.

In terms of departmental distribution, the Department of Nephrology (36.46%) was the high-incidence area for infections. This phenomenon is mainly due to the fact that some patients undergo long-term invasive procedures such as hemodialysis and central venous catheterization, leading to impaired skin and mucosal barriers, which provide a natural window for the adhesion and colonization of *S. aureus* (28, 29). This is highly consistent with the characteristics reported in an 18-year study on BSIs in hemodialysis patients in Switzerland by Hassoun-Kheir et al. (30), which confirmed that hemodialysis patients are a high-risk group for BSIs, and invasive hemodialysis access such as central venous catheters is one of the core factors leading to increased infection risk. Notably, *S. aureus* isolates detected in this study generally carried adhesion and biofilm-forming genes such as *fnb*, *ica*, and *sdr*, and dominant clones such as ST59 were often enriched with these genes. It is speculated that epidemic strains in the Department of Nephrology enhance their colonization stability on dialysis equipment and catheter surfaces by improving biofilm-forming ability, thereby increasing the risk of BSIs.

Clonal lineage analysis showed that ST59 (21.88%) was the dominant ST type in the hospital and most prevalent in 2020, while ST22 (13.54%) was a persistently epidemic clone (detected continuously over 5 years). The predominance of the ST59 clone observed in this study aligns with reports in the literature. ST59 has been reported as the most prevalent clone of CA-MRSA in Asia (31). This finding is consistent with our results, together confirming the epidemiological trend of ST59 as the dominant CA-MRSA clone in China. The detection proportion of ST59 in this hospital (21.88%) was higher than the 8.9% reported in that study, which is speculated to be related to the higher local epidemic intensity caused by cross-scenario transmission characteristics such as outpatient dialysis patients and healthcare worker cross-carriage in this hospital (32). As a typical CA-MRSA clone, the high detection rate of ST59 combined with the distribution of resistance elements dominated by SCCmec type IV (58.93%) confirms that CA-MRSA has become the core pathogenic population of BSIs in this hospital, further verifying the trend of “community-hospital” cross-border transmission of MRSA and explaining the sporadic distribution of strains across different departments.

The antimicrobial susceptibility results in this study showed a high consistency with antimicrobial resistance genomic analysis. 98.96% of *S. aureus* strains were resistant to penicillin G, and the detection rate of *blaZ*_79, the most important resistance gene, reached 43.75%. The β-lactamase encoded by this gene can hydrolyze penicillin-class antibiotics. Liang et al. reported a 100% penicillin resistance rate among 77 MRSA strains from 3 hospitals in Shaoxing, with a high consistency between susceptibility results and genotyping analysis, which is similar to the results of our study (33). The core resistance mechanism of MRSA is the carriage of the *mecA* gene. A total of 59 *mecA*-positive strains were identified in this study, among which *mecA*_6 (38/59) was dominant and closely associated with SCCmec elements. This molecular characteristic leads to the deregulation of *mecA* gene expression, allowing strains to maintain persistent methicillin-resistant phenotypes. In contrast, all type II strains (11.86%) carried the complete *mecI*-*mecR1*-*mecA* complex, which explains the differences in resistance phenotypes among strains with different SCCmec types (34). Notably, SCCmec typing showed that type IV (58.93%) was the main type, and all type IV and V strains lacked the *mecI* gene and retained only the *mecR1-mecA* structure. This finding aligns with recent epidemiological shifts. As reported by Hamada et al., in their molecular typing study of MRSA from bloodstream infections (2015–2017), the detection rate of SCC*mec* type II declined significantly from 60.7 to 20.6%, while that of type IV increased substantially from 32.1 to 73.5%, thereby replacing type II as the dominant clone (35). This result strongly supports that the high prevalence of SCCmec type IV observed in our study is not an isolated phenomenon, but rather reflects a broader trend of CA-MRSA genotypes increasingly replacing traditional HA-MRSA as important pathogens in healthcare settings. The vancomycin resistance of strain A12-22 isolated from the Department of Hepatobiliary and Pancreatic Surgery may be related to the carriage of the VanA operon, and such mutations can reduce the binding affinity of vancomycin to peptidoglycan (36). The high tigecycline resistance of strain A200-20 isolated from the Department of Oncology may originate from specific mutations in the carried *mepA* efflux pump gene, which synergizes with mutations in the ribosomal S10 protein and transcriptional repressors to collectively enhance bacterial drug efflux capacity (37).

Resistance gene co-occurrence analysis showed that the single presence of *blaZ*_79 (12 strains) and the combination of *mecA*_6/*blaZ*_29/*erm*(C)_18 (11 strains) were the main types, and *mec* family genes often coexisted with *bla* family genes. *Mec* genes are located on SCCmec elements, while *bla* genes are mostly carried on plasmids. Their synergistic transmission enables strains to acquire resistance to both penicillins and methicillin (38). The frequent co-occurrence of mec- and bla-family resistance genes observed in this study, likely facilitated by the synergistic transmission of genes located on SCCmec elements and plasmids, mediates the simultaneous acquisition of resistance to both penicillin and methicillin-class antibiotics, posing significant challenges for clinical management and infection control. The carriage rate of the *erm*(C)_13 gene among tested MRSA strains was 35.42%, which was significantly associated with the high resistance phenotypes of strains to erythromycin (68.75%) and clindamycin (66.67%), and is a key gene mediating high-level resistance to macrolide and lincosamide antibiotics (39). The discordant phenotype of strain A215-20 (“oxacillin-susceptible but cefoxitin-positive”) was confirmed as MRSA after re-verification. Its molecular mechanism may be related to the carriage of pseudo-SCCmec elements (3 pseudo-SCCmec strains were detected in this study). Pseudo-SCCmec elements lack complete *ccr* genes or regulatory regions of the mec complex, leading to low-level expression of the *mecA* gene, which cannot be detected by routine oxacillin susceptibility testing. However, cefoxitin can activate *mecA* expression, resulting in a positive result (40).

MRSA strains in this study carried an average of 74 virulence genes, among which 45 core virulence genes (related to adhesion, immune evasion, toxins, and enzymes) were present in all strains. Meanwhile, MRSA strains carried a high proportion of resistance genes, forming a synergistic characteristic of “high virulence-high resistance”. The widespread presence of adhesion and biofilm-forming genes (such as *fnb*, *ica*, *sdr*, *clf*) provides a molecular basis for strains to colonize host tissues and medical device surfaces, which is also an important strain-level reason for the high incidence of infections in the Department of Nephrology (41, 42). The extensive detection of leukocidin genes *lukS*-PV (13.54%) and *lukF*-PV (59.38%)—toxins that can disrupt neutrophil function and enhance bacterial immune evasion ability—is closely associated with the tendency of severe community-acquired infections (43). In addition, the detection of toxin genes such as *tsst*-1 (11.46%) and *sea*/*seb* (26.04%) suggests that some strains have the potential to cause special infections such as toxic shock syndrome and food poisoning (44, 45).

Based on the findings of this study, vancomycin remains the first-line agent for MRSA infections in accordance with major clinical guidelines, given the extremely low vancomycin resistance rate among *S. aureus* isolates in our cohort. Linezolid may be considered a valuable alternative for the treatment of MRSA infections in the Department of Nephrology and the Intensive Care Unit. Meanwhile, continued vigilance against vancomycin and tigecycline resistance is warranted in departments including the Department of Hepatobiliary and Pancreatic Surgery and the Department of Oncology. The Department of Nephrology should be designated as a key prevention and control ward, with strengthened measures for equipment disinfection, catheter care, and patient screening. WGS should be applied for transmission tracing of dominant clones such as ST59 and ST22. In addition, the irrational use of macrolides and quinolones should be strictly controlled to reduce the selective pressure for the emergence and persistence of corresponding resistance determinants.

This study has several limitations. First, as a single-center retrospective study, it was limited by a relatively small sample size (*n* = 96) and focused only on BSI-derived strains without incorporating skin and mucosal colonizing strains for comparison, which may introduce selection bias in depicting the nosocomial *S. aureus*. Second, the absence of detailed clinical outcome data (e.g., underlying diseases, treatment response, mortality) precluded an in-depth analysis of the association between the virulence gene profiles of dominant clones (such as ST59) and infection severity or patient prognosis. Furthermore, the functional verification of the identified pseudo-SCCmec elements was insufficient, and the specific mechanism regulating *mecA* expression remains unclarified. Future studies should involve multicenter prospective designs, integrate data from both colonizing and infecting strains, incorporate clinical outcome metrics, and employ *in vitro* experiments to validate the molecular mechanisms of special resistant strains and pseudo-SCCmec-carrying strains to provide more comprehensive evidence for the prevention and control of MRSA infections.

## 5 Conclusion

By integrating clinical epidemiological data and WGS results of *S. aureus* from BSIs in a tertiary hospital in central China between 2020 and 2024, this study showed that the prevalence of *S. aureus* from BSIs in the hospital was on a downward trend, with MRSA as the dominant subtype (72.92%), ST59 as the absolute dominant clone, and SCCmec type IV as the main resistance element. These findings confirm that CA-MRSA is the core pathogenic population of nosocomial BSIs, with the characteristic of “community-hospital” cross-border transmission. This study supplements the baseline data on nosocomial MRSA prevalence in central China, verifies the application value of WGS in nosocomial infection prevention and control, provides support for establishing a model of “genotypic surveillance-targeted prevention and control-precise medication”, and has important reference significance for similar institutions.

## Data Availability

The datasets presented in this study can be found in online repositories. The names of the repository/repositories and accession number(s) can be found in the article/Supplementary material.

## Footnotes

1
https://github.com/s-andrews/FastQC.git

2
https://github.com/OpenGene/fastp.git

3
https://github.com/ablab/quast.git

4
https://github.com/Ecogenomics/CheckM.git

5
https://github.com/tseemann/abricate

6
https://github.com/tseemann/mlst.git

7
https://github.com/tseemann/snippy.git

8
https://github.com/morgannprice/fasttree.git
